# Sex-specific CT-derived muscle and fat phenotypes in colon cancer: implications for nutritional and metabolic assessment

**DOI:** 10.3389/fnut.2025.1728741

**Published:** 2025-11-21

**Authors:** Fernanda Mucarzel, Patricia Guirado Peláez, Virginia Soria Utrilla, Rocío Fernández Jiménez, Fiorella Palmas Candia, Francisco José Sánchez Torralvo, Rosa Burgos Peláez, José Manuel García Almeida, Gabriel Olveira

**Affiliations:** 1 Nutrition Support Unit, Endocrinology and Nutrition Department, Hospital Universitari Vall d’Hebron, Barcelona, Spain; 2 Department of Endocrinology and Nutrition, Virgen de la Victoria University Hospital, Málaga, Spain; 3 Department of Endocrinology and Nutrition, Quironsalud Málaga Hospital, Málaga, Spain; 4 Unidad de Gestión Clínica de Endocrinología y Nutrición, Hospital Regional Universitario de Málaga, Málaga, Spain; 5 CIBEROBN, Carlos III Health Institute (ISCIII), University of Málaga, Málaga, Spain; 6 Instituto de Investigación Biomédica de Málaga y Plataforma en Nanomedicina-IBIMA Plataforma BINAND, Málaga, Spain; 7 Department of Medicine and Dermatology, Málaga University, Málaga, Spain; 8 Diabetes and Metabolism Research Unit, Vall d’Hebron Institut de Recerca (VHIR), Barcelona, Spain; 9 Department of Medicine, Universitat Autònoma de Barcelona, Barcelona, Spain

**Keywords:** computed tomography, colorectal cancer, body composition, Hounsfield units, postoperative complications

## Abstract

**Background:**

Computed Tomography (CT)-derived analysis of Body Composition (BC) provides detailed phenotyping of skeletal muscle and adipose tissue, offering insight into nutritional and metabolic risk in oncology.

**Objective:**

To characterize sex- and age-specific muscle and adipose tissue phenotypes in patients with colon cancer and describe their patterns across postoperative outcomes.

**Methods:**

Multicentric observational cross-sectional study including Colorectal Cancer (CRC) patients undergoing laparoscopic elective surgery. Preoperative CT scans at L3 were analyzed for muscle and adipose tissue quantity and radiodensity. Differences in BC parameters between patient groups (according to presence of complications, hospital stay and disease stage by sex) were assessed using Student’s *t*-test (*p* < 0.05).

**Results:**

502 CRC patients, predominantly males (62.5%) with a mean age of 68.08 ± 10.62 were included. Sex-specific differences in muscle quality and adipose tissue distribution were observed across postoperative outcomes. In women, lower Subcutaneous Adipose Tissue (SAT) values were observed in those with longer hospital stay and complications (*p* < 0.001), whereas in men, reduced Skeletal Lean Muscle Radiodensity (*p* < 0.001) and higher Visceral Adipose Tissue (VAT; *p* = 0.013) were found in those with adverse outcomes. These differences were independent of BMI and age.

**Conclusion:**

CT-based body composition phenotyping identifies distinct metabolic profiles linked to postoperative risk. Incorporating tissue quality and distribution into nutritional assessment may enhance early identification of vulnerable patients and guide personalized perioperative strategies.

## 1 Introduction

Body mass index (BMI) is widely used as a surrogate marker of nutritional status in oncology patients, primarily due to its simplicity, accessibility, and established associations with clinical outcomes (1). However, it does not distinguish between fat mass and fat-free mass compartments, and may therefore mask muscle depletion or malnutrition in individuals with a normal or elevated BMI (2). As a result, Body Composition (BC) assessment has emerged as a more accurate approach, providing detailed information on skeletal muscle mass quantity and quality, and, depending on the technique, adipose tissue distribution (3–7).

Malnutrition, as defined by the Global Leadership Initiative on Malnutrition (GLIM) criteria, is diagnosed when at least one phenotypic criterion (weight loss, low BMI or reduced skeletal muscle mass) is present along with one etiological criterion (8). However, universally accepted cutoffs for defining moderate versus severe muscle mass loss –adjusted for sex, age and disease—remain limited (9).

In this context, the concept of sarcopenia, as defined by the European Working Group on Sarcopenia in Older People 2 (EWGSOP2) becomes relevant: it is considered probable when low muscle strength is identified and confirmed when reduced muscle quantity is also demonstrated (10).

Among the types of cancer where Computed Tomography (CT) imaging is systematically used for diagnosis, ColoRectal Cancer (CRC) represents a particularly robust model for BC research in surgical outcomes (11). However, most previous studies have combined patients undergoing laparoscopic and open procedures, which may introduce heterogeneity in postoperative outcomes (12–14).

The routine use of preoperative abdominal CT allows for opportunistic assessment of BC. In particular, CT images at the L3 vertebral level provide highly accurate and reproducible measures not only of tissue quantity but also of radiodensity and distribution, allowing for detailed phenotypic characterization (12, 13).

Low Skeletal Muscle Index (SMI) has been linked to adverse outcomes in multiple types of cancers (14–20). However, proposed cut-offs for CT-derived measurements—such as those by Prado et al. or Martin et al.—are based mainly on muscle indices and mortality risk, and may not fully capture the complexity of perioperative outcomes (19, 21–24).

Most studies to date have not adequately considered the inherent differences in body composition based on sex, age, or tumor stage, limiting the generalizability of the proposed cutoff values. Addressing these shortcomings is essential to ensure that CT-derived parameters, such as low SMI or reduced skeletal muscle radiodensity (SMD), truly reflect clinically meaningful malnutrition, sarcopenia, or myosteatosis in the perioperative setting (25–29).

Sex-related differences in body composition are well established: women generally exhibit higher Subcutaneous Adipose Tissue (SAT), whereas men tend to accumulate more Visceral Adipose Tissue (VAT) and greater absolute muscle mass (30). These distinct phenotypes may modulate inflammatory and metabolic responses and influence surgical recovery, but their clinical significance in homogeneous cohorts of CRC patients remains insufficiently defined. Furthermore, aging is accompanied by progressive declines in both muscle mass and muscle quality, adding another layer of variability to risk stratification (31–33).

Based on these considerations, the present study aimed to characterize sex-specific BC phenotypes in patients with colon cancer undergoing surgery, using CT-derived measurements of muscle and adipose compartments. We further examined differences in these phenotypes according to length of hospital stay as well as the presence of postoperative complications, and explored how age and tumor stage might influence these patterns.

## 2 Materials and methods

### 2.1 Study design

This was a multicenter, cross-sectional study conducted in accordance with the Declaration of Helsinki between October 2018 and July 2024 at the Endocrinology and Nutrition services of the Regional University Hospital of Malaga, Virgen de la Victoria Hospital of Malaga, and Vall d’Hebron Hospital of Barcelona. The protocol was approved by the Research Ethics Committee for Medicines of Vall d’Hebron University Hospital on February 29, 2024 (reference PR(AG)489/2021), covering inter-hospital data sharing and retrospective analysis of patient data. Data from 171 patients from Vall d’Hebron Hospital, 123 from Virgen de la Victoria Hospital, and 208 from the Regional University Hospital of Malaga were included. An internal protocol was followed to standardize data collection procedures across centers, ensuring consistency in the methodology and the use of comparable equipment for all assessments.

The sample was considered representative, as patients were consecutively recruited from three tertiary public hospitals with heterogeneous populations. With *n* = 502, the maximum margin of error for estimating a prevalence (95% CI, *p* = 0.5) is ±4.4%, ensuring adequate precision. For group comparisons of similar size (≈251 per group), the study has 80% power to detect small-to-moderate differences (Cohen’s d ≈ 0.25, *α* = 0.05).

### 2.2 Patient selection

For all three hospitals, eligible participants were adults (≥18 years) with histologically confirmed colorectal cancer (CRC) scheduled for elective laparoscopic surgery, with availability of a diagnostic or staging abdominal CT performed < 30 days prior to surgery. All participants were at risk of malnutrition according to the Malnutrition Universal Screening Tool (MUST) and provided written informed consent.

Ineligibility criteria included urgent or non-laparoscopic procedures, lack of lumbar coverage or partial loss of the analyzable area, and image-quality limitations in the region of interest (e.g., metallic prostheses, beam hardening, attenuation distortion, or excessive noise) precluding reliable body-composition assessment.

### 2.3 Clinical data collection

Data acquisition was centralized and coordinated by the Vall d’Hebron team to ensure uniform procedures and quality control across centers. All participants underwent medical history, anthropometry, nutritional assessment, and abdominal CT. Oncological variables included tumor location, and cancer stage was assigned according to the American Joint Committee on Cancer (AJCC) staging system version 9 (34).

### 2.4 Anthropometric and nutritional assessment

The following variables were systematically recorded by trained personnel at each hospital: height in meters, weight in kg, Body Mass Index (BMI) in kg/m^2^, as well as weight loss in the previous 6 months (%). Fat Free Mass Index in Kg/m^2^ (FFMI) was assessed by Bioelectrical Impedance Analysis (BIA) in all participating institutions using the same portable device (Akern BIA-101/Nutrilab analyzer, Akern SRL, Pontassieve, Florence, Italy). The technical accuracy of the instrument was verified daily with a precision check provided by the manufacturer. This standardized approach minimized inter-hospital variability and ensured measurement consistency across centers.

Nutritional risk was screened with Malnutrition Universal Screening Tool (MUST) as the first-step screening tool (35). The diagnosis of malnutrition was made following Global Leadership Initiative on Malnutrition (GLIM) criteria (8).

It was estimated that all patients presented at least one etiological criterion, as they had colorectal neoplasia (considered a chronic inflammatory process). For the phenotypic criterion of reduced muscle mass, patients were classified using the Fat-Free Mass Index (FFMI) according to the cutoff points (<17 kg/m^2^ for men and <15 kg/m^2^ for women) recommended by the consensus itself as measured by BIA (9).

### 2.5 CT body composition analysis

To assess skeletal muscle and abdominal adipose tissue area, transverse CT images at the level of the third lumbar vertebra (L3) were analyzed using the FocusedON-BC software Version 2.1. These images were acquired with a multidetector CT scanner and DICOM files were provided by the Radiology Department of the participating hospitals. Tissue segmentation was performed by trained personnel at each center, employing the semi-automated tool integrated within the FocusedON-BC software to identify and quantify body composition compartments.

The abdominal muscle groups analyzed included the psoas, erector spinae, quadratus lumborum, transversus abdominis, external and internal obliques, and rectus abdominis. Adipose tissue was categorized into subcutaneous, visceral, and intermuscular compartments (36, 37).

**Figure 1:**
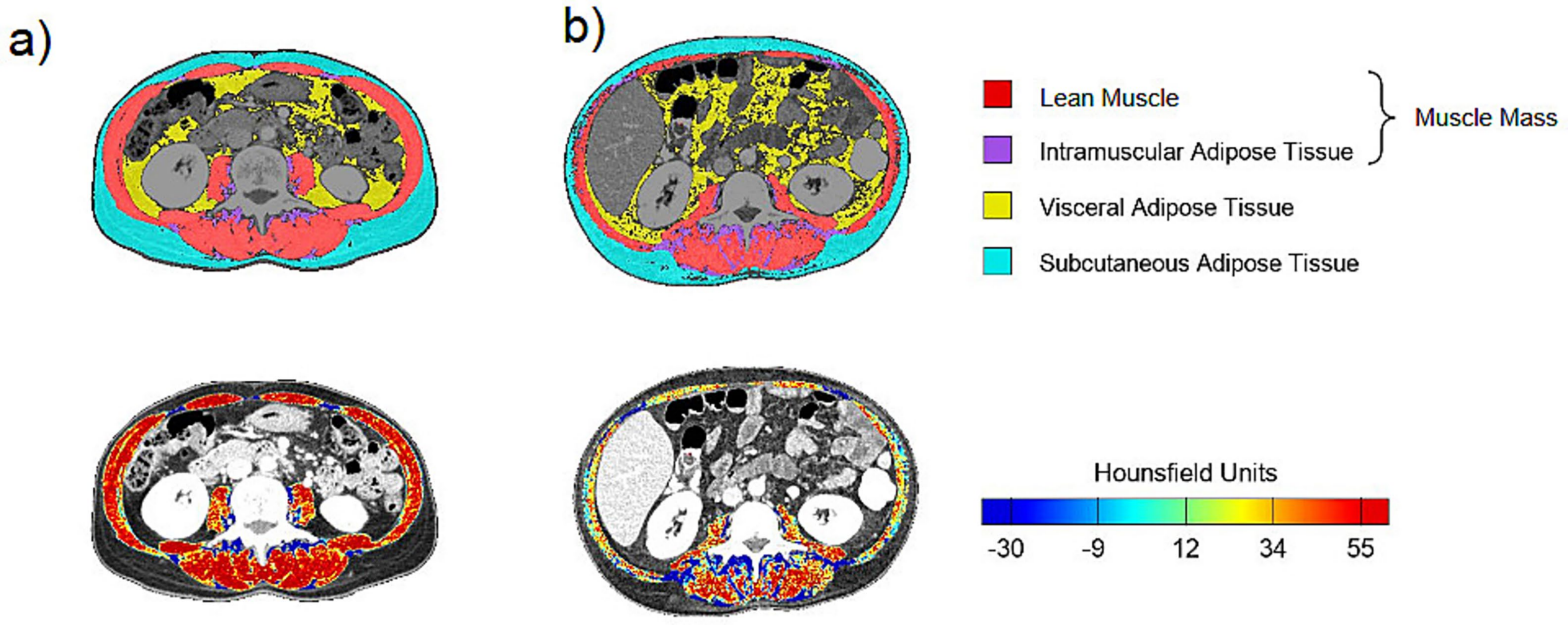
Images extracted from the FocusedON-BC software: patients **(a)** and **(b)** presented the same BMI (30.7 kg/m^2^), but different LMMA percentages (22.84 and 14.25%, respectively) and lean muscle radiodensity (50.61 HU and 34.46 HU, respectively).

Unlike conventional CT analysis software, which typically assesses muscle mass without distinguishing between lean muscle mass and intermuscular adipose tissue, FocusedON-BC software allows for segmentation of those tissues, providing a more detailed and precise assessment (Figure 1). However, for comparability with previously published data, both conventional and detailed segmentation approaches were applied in this study.

The variables recorded included Skeletal Muscle Area (SMA, in cm^2^ and %), Skeletal Muscle Index (calculated by SMA normalized to the patient’s height squared (cm^2^/m^2^), SMI in cm^2^/m^2^), Lean Muscle Mass Area (LMMA, in cm^2^ and %), Lean Muscle Mass Index (LMMI, in cm^2^/m^2^), InterMuscular Adipose Tissue area (IMAT, in cm^2^ and %) and Index (IMATI, in cm^2^/m^2^), Visceral Adipose Tissue area (VAT, in cm^2^ and %) and Index (VATI, in cm^2^/m^2^), and, Subcutaneous Adipose Tissue area (SAT, in cm^2^ and %) and Index (SATI, in cm^2^/m^2^).

Tissue quality for muscle mass was assessed based on its radiodensity (D) measured by mean of Hounsfield Units (HU), applying standard radiodensity thresholds: −29 to 150 HU for skeletal muscle mass, −190 to −30 for SAT and −150 to −50 for VAT (24).

### 2.6 Surgical outcomes

Short-term outcomes comprised length of stay (recorded in days), postoperative complications within 30 days from surgery (presence or absence), and discharge destination. Length of stay was categorized according to previously published criteria (38), with hospitalizations longer than 10 days indicating poorer prognosis and stays shorter than 6 days considered as expected recovery. Postoperative complications were further classified according to the Clavien–Dindo criteria (39). Furthermore, discharge destination was recorded as home, convalescence center, home hospitalization program, or in-hospital death.

Readmission during the 3-month follow-up period and mortality during the 6-month follow-up after discharge were also documented and analyzed to assess long-term surgical outcomes.

### 2.7 Statistical analysis

Statistical analysis was performed using Python 3.11 (Python Software Foundation).^1^

Continuous variables are presented as mean ± standard deviation (SD) for normal distributed variables and median ± interquartile range (IR) for non-normal distributed variables. Categorical variables are presented as frequencies and percentages, n (%). Statistical significance was accepted at *p* < 0.05.

Kolmogorov–Smirnov test was used to assess the normal distribution of the dataset.

Differences between groups were assessed using an independent samples t-test for normally distributed variables, the non-parametric Mann–Whitney U test for non-normally distributed variables and the X^2^ test for categorical variables.

## 3 Results

### 3.1 Study population

A total of 502 patients with Colorectal Cancer (CRC) undergoing elective laparoscopic surgery were included (Table 1). The cohort was predominantly men (63%), with a mean age of 68.08 ± 10.62 years, with no significant sex-related differences (*p* = 0.457).

**Table 1:** General features of the sample.

Baseline characteristics		Total (*n* = 502)	Men (*n* = 314)	Women (*n* = 188)	*p* value
Age (years)	m ± SD	68.08 ± 10.62	67.8 ± 10.9	67.8 ± 10.9	0.457
Stage	n(%)				
I		94 (19.9)	64 (21.9)	30 (16.8)	
II		154 (32.7)	97 (33.2)	57 (31.8)	
III		184 (39.1)	105 (35.9)	79 (44.2)	
IV		39 (8.3)	26 (9.0)	13 (7.2)	0.306
Tumor location	n(%)				
Colon		357 (71.0)	221 (70.0)	136 (72.0)	
Rectum		145 (29.0)	93 (30.0)	52 (28.0)	0.714
Post-operative complications	n(%)	159 (32.0)	109 (35.0)	50 (27.0)	0.073
GLIM criteria, malnourished (8)	n(%)	200 (40)	114 (36)	86 (46)	**0.046**
BMI (kg/m^2^)	Med[IR]	26.62 [23.8, 29.5]	26.9 [24.13, 29.29]	26.2 [22.95, 29.6]	0.308
Previous 6 months weight loss, % (*n* = 274)	Med[IR]	1.7 [0.0, 6.43]	1.68 [0.0, 6.1]	2.07 [0.0, 7.32]	0.219
FFMI kg(m^2^)	n(%)	19.41 ± 2.88	20.58 ± 2.57	17.52 ± 2.3	**<0.001**

BMI, Body Mass Index; m, mean; SD, Standard Deviation; med (IR), median (Interquartile Range); FFMI, Fat Free Mass Index. Bold values indicate a significant *p*-value <0.05.

According to GLIM criteria, 40% presented malnutrition (36% men and 46% women, *p* = 0.046) despite the average BMI was between the normal range (BMI 26.62 kg/m2 [23.8–29.5]) according to age, without differences by sex (*p* = 0.308) (38).

Sex-specific differences were observed in the Fat-Free Mass Index (FFMI), with women presenting significantly lower values than men (*p* < 0.001), as expected according to the cut-off points for low muscle mass. Disease staging was not markedly advanced, with 92% of patients classified as stage ≤3, without differences by sex (*p* = 0.306).

Overall complication rates were comparable (35% in men vs. 27% in women; *p* = 0.073), although men appeared to experience a higher incidence of postoperative complications.

Subgroup analyses are presented below, performed as required to address the findings observed.

### 3.2 Body composition assessment through CT prior to surgery

CT-derived preoperative body composition parameters stratified by sex are shown in Table 2. Distinct sex-related phenotypes were observed.

**Table 2:** CT body composition parameters by sex prior to surgery.

CT-derived BC		Men (*n* = 314)	Women (*n* = 188)	*p* value
Lean muscle	LMMA (cm^2^)	131.12 ± 25.55	91.11 ± 16.06	**<0.001**
	LMMA (%)	17.64 ± 4.43	14.52 ± 3.97	**<0.001**
	LMMI (cm2/m^2^)	45.36 ± 8.47	36.74 ± 6.09	**<0.001**
	LMD (HU)	39.69 ± 9.19	36.93 ± 9.97	**0.002**
Intermuscular adipose tissue	IMAT (cm^2^)	13.3 [8.66, 21.46]	13.56 [9.17, 20.42]	0.996
	IMAT (%)	1.8 [1.22, 2.8]	2.09 [1.46, 2.99]	**0.007**
	IMATD (HU)	−63.5 ± 6.55	−63.53 ± 6.54	0.963
	IMATI (cm2/m^2^)	4.65 [3.03, 7.57]	5.58 [3.66, 8.44]	**0.014**
Skeletal muscle	SMA (cm2)	147.76 ± 25.89	107.01 ± 18.61	**<0.001**
	SMA (%)	19.23 [16.86, 22.17]	16.41 [14.16, 18.73]	**<0.001**
	SMI (cm^2^/m^2^)	51.15 ± 8.71	43.17 ± 7.03	**<0.001**
	SMD (HU)	28.28 ± 14.22	22.55 ± 14.92	**<0.001**
Visceral adipose tissue	VAT (cm^2^)	176.31 [124.19, 259.45]	169.24 [102.1, 232.19]	**0.041**
	VAT (%)	23.36 [17.12, 30.64]	25.16 [17.34, 32.41]	0.25
	VATD (HU)	−95.2 [−100.09, −90.41]	−96.65 [−101.12, −91.01]	0.11
	VATI (cm^2^/m^2^)	59.54 [43.2, 89.16]	67.8 [42.26, 93.52]	0.404
Subcutaneous adipose tissue	SAT (cm^2^)	172.51 [122.23, 249.03]	164.44 [99.07, 259.75]	0.163
	SAT (%)	22.8 [17.48, 29.44]	24.95 [16.9, 33.91]	0.125
	SATD (HU)	−95.54 [−100.77, −90.12]	−96.76 [−102.96, −88.28]	0.696
	SATI (cm^2^/m^2^)	58.71 [42.33, 84.54]	65.88 [40.62, 104.59]	0.235

CT, Computed Tomography; HU, Hounsfield Units; LMMA, Lean Muscle Mass Area; LMMI, Lean Muscle Mass Index; LMD, Lean Muscle Radiodensity; IMAT, InterMuscular Adipose Tissue; IMATD, InterMuscular Adipose Tissue Radiodensity; IMATI, InterMuscular Adipose Tissue Index; SMA, Skeletal Muscle Area; SMI, Skeletal Muscle Index; VAT, Visceral Adipose Tissue; VATD, Visceral Adipose Tissue radiodensity; VATI, Visceral Adipose Tissue Index; SAT, Subcutaneous Adipose Tissue; SATD, Subcutaneous Adipose Tissue Radiodensity; SATI, Subcutaneous Adipose Tissue Index. Results are expressed as mean ± standard deviation or median [interquartile range]. Bold values indicate a significant *p*-value <0.05.

Men exhibited significantly higher absolute and indexed skeletal muscle areas compared with women, including LMMA (131.12 ± 25.55 cm^2^ vs. 107.01 ± 18.61 cm^2^, *p* < 0.001), SMA (147.76 ± 25.89 cm^2^ vs. 107.01 ± 18.61 cm^2^, *p* < 0.001), and their respective indices (LMMI and SMI, both *p* < 0.001). They also demonstrated higher Lean Muscle Radiodensity (LMD) values (39.69 ± 9.19 HU vs. 36.93 ± 9.97 HU, *p* = 0.002) as well as Skeletal Muscle Radiodensity (SMD; *p* < 0.001), indicating better muscle quality at same age (*p* = 0.457).

In contrast, women showed a higher IMAT percentage compared to men (2.09% vs. 1.80%, *p* = 0.007) and standardized IMAT index (IMATI) was also greater in women (5.58 cm^2^/m^2^ [3.66–8.44] vs. 4.65 cm^2^/m^2^ [3.03–7.57], *p* = 0.014). On the other side, VAT area was higher in men (176.31 cm^2^ vs. 169.24 cm^2^; *p* = 0.041).

Nevertheless, no statistically significant sex differences were observed regarding adipose tissue radiodensity in the studied sample.

### 3.3 Postoperative outcomes by sex

Postoperative outcomes by sex are further summarized in Table 3.

**Table 3:** Post-operative outcomes by sex.

Post-operative outcomes		Men (*n* = 314)	Women (*n* = 188)	*p* value
Hospital stay				
Length of stay (days)	Med[IR]	7 [4.0, 12.0]	6 [5.0, 8.0]	0.077
< 6 days (*n* = 250)	n(%)	145 (60)	105 (73)	
≥ 10 days (*n* = 134)		97 (40)	37 (26)	**0.008**
Discharge destination (*n* = 294)*	n(%)			
Home		175 (93)	103 (97)	
CSC		5 (3)	2 (2)	
PHD		4 (2)	1 (1)	
Exitus during hospital stay		4 (2)	0 (0)	0.184
Post-operative complications				
Clavien Dindo (35) ≥ 3	n(%)	34 (11)	7 (4)	**0.008**
Hospital readmission	n(%)	33 (11)	12 (6)	0.16
Mortality at 6 months	n(%)	25 (8)	12 (6)	0.632

CSC, convalescence center; PHD, Home hospitalization program; IR, interquartile range; *Post-hoc comparison did not show a significant difference by sex in discharge destination between home and exitus (*p* = 0.549), neither between other groups. Bold values indicate a significant *p*-value <0.05.

Median hospital stay was slightly longer in men (7 [4.0–12.0] days) than in women (6 [5.0–8.0] days), although not statistically significant (*p* = 0.077). However, women were more frequently discharged within < 6 days (73% vs. 60%; *p* = 0.008) and men more frequently discharged after 10 days (40% vs. 26%; *p* = 0.008). Discharge destination was predominantly home across sex without significant variation among destinations such as convalescence centers (*p* = 0.184). Readmission rates were similar across sex (*p* = 0.16).

Considering that overall complication rates were comparable by sex, (35% in men vs. 27% in women; *p* = 0.073), major complications (Clavien–Dindo ≥3) were significantly less frequent in women (4% vs. 11%; *p* = 0.008).

The 6-month postoperative mortality rate in the cohort was 7%, without significant sex-related differences (*p* = 0.632).

### 3.4 Body composition and clinical outcomes in men

#### 3.4.1 BC in men considering length of hospital stay

A higher proportion of men experienced prolonged hospital stay (>10 days) compared to women. Therefore, body composition characteristics were further analyzed according to length-of-stay categories (Table 4).

**Table 4:** Men body composition parameters by CT by hospital stay.

*n* = 242		< 6 days (*n* = 145)	≥ 10 days (*n* = 97)	*p* value
CT BC parameters				
Lean muscle	LMMA (cm^2^)	134.33 ± 26.26	126.0 ± 25.64	**0.015**
	LMMA (%)	18.0 ± 16.45	16.45 ± 3.5	**0.0041**
	LMMI (cm2/m^2^)	46.13 ± 8.77	43.9 ± 8.52	0.051
	LMD (HU)	41.74 ± 9.52	36.27 ± 7.92	**<0.001**
Intermuscular adipose tissue	IMAT (cm^2^)	13.24 [8.72, 21.41]	15.03 [9.59, 22.56]	0.342
	IMAT (%)	1.79 [1.23, 2.83]	1.91 [1.23, 2.83]	0.58
	IMATD (HU)	−65.3 ± 6.78	−62.34 ± 5.53	**<0.001**
	IMATI (cm2/m^2^)	4.64 [3.26, 8.1]	6.05 [3.97, 8.77]	**0.025**
Visceral adipose tissue	VAT (cm^2^)	207.56 ± 110.79	189.56 ± 100.76	**<0.001**
	VAT (%)	26.02 [18.84, 32.05]	21.73 [16.59, 29.73]	**0.017**
	VATD (HU)	−96.03 [−101.07, −91.78]	−94.36 [−98.66, −90.12]	0.052
	VATI (cm^2^/m^2^)	67.27 [45.04, 95.98]	56.82 [43.75, 85.49]	0.257
Subcutaneous adipose tissue	SAT (cm^2^)	163.53 [120.25, 220.81]	198.65 [132.69, 262.73]	**0.017**
	SAT (%)	22.09 [17.84, 27.62]	24.25 [18.55, 33.58]	**0.016**
	SATD (HU)	−97.64 ± 9.25	−92.55 ± 10.61	**<0.001**
	SATI (cm^2^/m^2^)	54.41 [41.14, 76.85]	67.82 [46.68, 99.88]	**0.007**
Demographic and clinical data				
Age		67.51 ± 10.91	69.9 ± 10.44	0.091
BMI		27.18 ± 4.27	26.99 ± 4.51	0.731
Weight loss	**(%)**	1.64 [0.0, 6.01]	4.4 [0.0, 9.31]	**0.035**
Malnutrition*	n(%)	43 (30)	45 (46)	**0.012**
Post-operative complications	n(%)	6 (4)	86 (89)	**<0.001**
Exitus	n(%)	7 (5)	13 (13)	**0.033**

CT, Computed Tomography; HU, Hounsfield Units; LMMA, Lean Muscle Mass Area; LMMI, Lean Muscle Mass Index; LMD, Lean Muscle Radiodensity; IMAT, InterMuscular Adipose Tissue; IMATD, InterMuscular Adipose Tissue Radiodensity; IMATI, InterMuscular Adipose Tissue Index; VAT, Visceral Adipose Tissue; VATD, Visceral Adipose Tissue radiodensity; VATI, Visceral Adipose Tissue Index; SAT, Subcutaneous Adipose Tissue; SATD, Subcutaneous Adipose Tissue Radiodensity; SATI, Subcutaneous Adipose Tissue Index. Results are expressed as mean ± standard deviation or median [interquartile range]. *GLIM criteria is used for diagnosis of malnutrition. Bold values indicate a significant *p*-value <0.05.

In this subgroup, men with a standard hospital stay (<6 days) consistently showed higher lean mass parameters, including LMMA and radiodensity, compared with those with a prolonged hospital stay (**≥**10 days), despite having comparable BMI (*p* = 0.731) and age (*p* = 0.091).

Visceral adipose tissue (VAT) was higher in men with a standard hospital stay, as indicated by a greater percentage of VAT (*p* < 0.017). In contrast, subcutaneous adipose tissue (SAT) compartments were increased among men hospitalized for more than 10 days, as reflected by higher SAT, SATD, and SAT values (all *p* < 0.005).

#### 3.4.2 BC in men considering presence of postoperative complications

Given their higher rate of severe postoperative complications (Clavien Dindo >3), men were further analyzed by complication status (Table 5).

**Table 5:** Men body composition parameters by CT by presence of post-operative complication.

Men (*n* = 314)		No post-op complication (*n* = 205)	Presence of post-op complication (*n* = 109)	*p* value
CT BC parameters				
Lean muscle	LMMA (cm^2^)	134.45 ± 24.91	124.85 ± 25.66	**0.001**
	LMMA (%)	18.08 ± 4.72	16.82 ± 3.7	**0.017**
	LMMI (cm2/m^2^)	46.3 ± 8.31	43.59 ± 8.51	**0.007**
	LMD (HU)	41.07 ± 9.19	37.1 ± 8.65	**<0.001**
Intermuscular adipose tissue	IMAT (cm^2^)	13.15 [8.41, 21.66]	14.49 [9.47, 21.13]	0.347
	IMAT (%)	1.77 [1.2, 2.8]	1.86 [1.29, 2.82]	0.279
	IMATD (HU)	−64.1 ± 6.68	−62.38 ± 6.17	**0.027**
	IMATI (cm2/m^2^)	4.58 [2.94, 7.58]	5.0 [3.23, 7.24]	0.247
Skeletal muscle	SMA (cm2)	150.67 ± 25.32	142.29 ± 26.17	**0.006**
	SMA (%)	20.21 ± 4.57	19.07 ± 3.34	**0.023**
	SMI (cm^2^/m^2^)	51.91 ± 8.56	49.73 ± 8.86	**0.034**
	SMD (HU)	29.94 ± 13.89	25.16 ± 14.37	**0.004**
Visceral adipose tissue	VAT (cm^2^)	184.4 [124.18, 278.11]	161.28 [122.67, 231.11]	0.071
	VAT (%)	24.65 [17.7, 31.64]	21.73 [16.64, 28.05]	**0.013**
	VATD (HU)	−95.4 [−100.74, −91.06]	−94.3 [−98.65, −89.89]	0.12
	VATI (cm^2^/m^2^)	64.46 [43.16, 95.27]	55.13 [43.07, 78.23]	0.109
Subcutaneous adipose tissue	SAT (cm^2^)	166.35 [120.12, 235.56]	187.01 [127.45, 260.38]	0.091
	SAT (%)	22.16 [17.38, 28.42]	25.37 [18.16, 32.86]	**0.023**
	SATD (HU)	−96.08 [−102.71, −91.58]	−94.35 [−99.3, −88.27]	**0.009**
	SATI (cm^2^/m^2^)	55.75 [41.14, 79.54]	65.08 [43.78, 93.49]	0.067
Demographic and clinical data				
Age		67.12 ± 10.95	69.08 ± 10.73	0.129
BMI		26.85 [24.23, 29.39]	26.95 [23.72, 29.04]	0.624
Stage ≥ 4	n(%)	13 (6)	13 (12)	0.051
Malnutrition*	n(%)	65 (32)	49 (45)	**0.028**
Hospital length		5 [3.0, 7.0]	15 [10.0, 22.0]	**<0.001**
Exitus	n(%)	10 (5)	15 (14)	**0.011**

CT, Computed Tomography; HU, Hounsfield Units; LMMA, Lean Muscle Mass Area; LMMI, Lean Muscle Mass Index; LMD, Lean Muscle Radiodensity; IMAT, InterMuscular Adipose Tissue; IMATD, InterMuscular Adipose Tissue Radiodensity; IMATI, InterMuscular Adipose Tissue Index; SMA, Skeletal Muscle Area; SMI, Skeletal Muscle Index; VAT, Visceral Adipose Tissue; VATD, Visceral Adipose Tissue radiodensity; VATI, Visceral Adipose Tissue Index; SAT, Subcutaneous Adipose Tissue; SATD, Subcutaneous Adipose Tissue Radiodensity; SATI, Subcutaneous Adipose Tissue Index. BMI, Body Mass Index. Results are expressed as mean ± standard deviation or median [interquartile range]. *GLIM criteria is used for diagnosis of malnutrition. Bold values indicate a significant *p*-value <0.05.

Those with complications showed significantly lower lean muscle mass (area, index, and radiodensity; all *p* < 0.01) and less favorable adipose tissue profiles (higher SAT percentage and radiodensity, lower VAT percentage; all *p* < 0.05).

Despite comparable age and BMI, men with complications had a markedly longer hospital stay (*p* < 0.001), a higher prevalence of GLIM-defined malnutrition (*p* = 0.028), and increased 6-month mortality (*p* = 0.011).

Overall, the body composition patterns observed in men across length-of-stay categories were similar to those seen in men who experienced postoperative complications.

#### 3.4.3 BC in men considering disease stage

Because men who presented postoperative complications included more patients with advanced-stage disease (12% vs. 6%; *p* = 0.051), body composition was further compared by stage (Table 6).

**Table 6:** Men body composition parameters by CT by disease stage.

Men (*n* = 188)		I-III stage (*n* = 174)	IV stage (*n* = 14)	*p* value
CT BC parameters				
Lean muscle	LMMA (cm^2^)	129.64 ± 26.18	123.51 ± 27.63	0.402
	LMMA (%)	17.55 ± 4.43	18.3 ± 4.11	0.54
	LMMI (cm2/m^2^)	44.69 ± 8.72	41.65 ± 9.04	0.212
	LMD (HU)	40.63 ± 9.77	39.98 ± 9.01	0.808
Intermuscular adipose tissue	IMAT (cm^2^)	13.63 [9.04, 24.04]	10.39 [6.36, 19.95]	0.142
	IMAT (%)	1.92 [1.29, 3.0]	1.68 [1.1, 2.28]	0.307
	IMATD (HU)	−65.64 ± 6.48	−62.9 ± 7.16	0.132
	IMATI (cm2/m^2^)	4.78 [3.22, 8.3]	3.35 [2.49, 6.76]	0.102
Skeletal muscle	SMA (cm2)	147.54 ± 25.95	138.84 ± 27.46	0.231
	SMA (%)	19.96 ± 4.29	20.35 ± 3.0	0.74
	SMI (cm^2^/m^2^)	50.88 ± 8.75	46.81 ± 8.84	0.096
	SMD (HU)	28.0 ± 15.24	28.84 ± 17.48	0.402
Visceral adipose tissue	VAT (cm^2^)	212.26 ± 112.45	159.0 ± 97.71	0.087
	VAT (%)	26.94 [18.76, 33.42]	22.9 [10.44, 30.17]	0.099
	VATD (HU)	−94.8 ± 7.94	−89.18 ± 9.82	**0.013**
	VATI (cm^2^/m^2^)	73.68 ± 40.2	53.28 ± 31.18	0.065
Subcutaneous adipose tissue	SAT (cm^2^)	160.8 ± 69.1	154.34 ± 73.01	0.738
	SAT (%)	20.82 [16.31, 24.66]	21.87 [15.66, 27.4]	0.692
	SATD (HU)	−98.41 [−104.81, −93.11]	−96.13 [−100.27, −79.57]	**0.05**
	SATI (cm^2^/m^2^)	52.71 [38.82, 67.86]	55.26 [29.77, 69.08]	0.067
Demographic and clinical data				
Age		68.63 ± 10.97	61.5 ± 10.99	**0.02**
BMI		26.89 ± 4.16	25.66 ± 3.74	0.286
Weight loss	**(%)**	1.68 [0.0, 6.1]	5.14 [1.85, 13.72]	**0.023**
Malnutrition*	n(%)	59 (34)	7 (50)	0.356
Post-operative complications	n(%)	36 (21)	6 (43)	0.114
Exitus	n(%)	15 (9)	3 (21)	0.137

CT, Computed Tomography; HU, Hounsfield Units; LMMA, Lean Muscle Mass Area; LMMI, Lean Muscle Mass Index; LMD, Lean Muscle Radiodensity; IMAT, InterMuscular Adipose Tissue; IMATD, InterMuscular Adipose Tissue Radiodensity; IMATI, InterMuscular Adipose Tissue Index; SMA, Skeletal Muscle Area; SMI, Skeletal Muscle Index; LMR, Lean Muscle Ratio; VAT, Visceral Adipose Tissue; VATD, Visceral Adipose Tissue radiodensity; VATI, Visceral Adipose Tissue Index; SAT, Subcutaneous Adipose Tissue; SATD, Subcutaneous Adipose Tissue Radiodensity; SATI, Subcutaneous Adipose Tissue Index; BMI, Body Mass Index. Results are expressed as mean ± standard deviation or median [interquartile range]. *GLIM criteria is used for diagnosis of malnutrition. Bold values indicate a significant *p*-value <0.05.

Men with advanced disease stage were younger (*p* = 0.02), reported greater preoperative weight loss (*p* = 0.023), exhibited a tendency of less quantity of AT, and significant higher adipose tissue radiodensity (subcutaneous and visceral), while muscle parameters remained similar between groups, as shown in Table 7.

**Table 7:** Women body composition parameters by CT according to length of hospital stay.

Women (=142)		< 6 days (*n* = 105)	≥ 10 days (*n* = 37)	*p* value
CT BC parameters				
Lean muscle	LMMA (cm^2^)	92.85 ± 16.59	93.61 ± 15.53	0.809
	LMMA (%)	14.29 ± 4.06	15.09 ± 3.81	0.296
	LMMI (cm2/m^2^)	36.94 ± 6.18	37.42 ± 6.3	0.686
	LMD (HU)	37.87 ± 9.79	35.4 ± 9.77	0.189
Intermuscular adipose tissue	IMAT (cm^2^)	14.25 [10.25, 20.98]	11.67 [9.02, 20.74]	0.247
	IMAT (%)	2.19 [1.51, 3.06]	2.04 [1.58, 2.91]	0.52
	IMATD (HU)	−64.95 ± 6.61	−62.41 ± 6.11	**0.043**
	IMATI (cm2/m^2^)	5.81 [4.01, 8.44]	4.76 [3.45, 8.35]	0.227
Skeletal muscle	SMA (cm2)	109.74 ± 20.01	108.52 ± 15.52	0.738
	SMA (%)	16.71 ± 3.97	17.33 ± 3.47	0.399
	SMI (cm^2^/m^2^)	43.66 ± 7.39	43.36 ± 6.01	0.823
	SMD (HU)	22.68 ± 14.52	22.19 ± 14.47	0.86
Visceral adipose tissue	VAT (cm^2^)	162.04 ± 82.53	196.19 ± 123.24	0.061
	VAT (%)	23.58 ± 11.44	27.68 ± 12.9	0.073
	VATD (HU)	−96.68 [−101.25, −90.88]	−97.83 [−101.32, −88.59]	0.9
	VATI (cm^2^/m^2^)	64.76 ± 33.1	77.82 ± 46.99	0.068
Subcutaneous adipose tissue	SAT (cm^2^)	213.13 ± 123.77	139.44 ± 93.41	**0.001**
	SAT (%)	28.89 ± 11.77	20.27 ± 10.09	**<0.001**
	SATD (HU)	−100.13 [−105.42, −93.35]	−92.14 [−97.25, −85.59]	**<0.001**
	SATI (cm^2^/m^2^)	85.25 ± 49.37	55.0 ± 36.58	**<0.001**
Demographic and clinical data				
Age		67.99 ± 9.58	67.86 ± 10.44	0.947
BMI		27.17 ± 5.41	26.77 ± 5.46	0.702
Tumor location	n(%)			
Colon		83 (79)	23 (62)	**0.07**
Rectum		22 (21)	14 (38)	
Stage ≥ 4	n(%)	6 (6)	3 (8)	0.602
Malnutrition*	n(%)	41 (39)	20 (54)	0.164
Post-op Complication	n(%)	9 (9)	30 (81)	**<0.001**
Exitus	n(%)	3 (3)	4 (11)	0.076

CT, Computed Tomography; HU, Hounsfield Units; LMMA, Lean Muscle Mass Area; LMMI, Lean Muscle Mass Index; LMD, Lean Muscle Radiodensity; IMAT, InterMuscular Adipose Tissue; IMATD, InterMuscular Adipose Tissue Radiodensity; IMATI, InterMuscular Adipose Tissue Index; SMA, Skeletal Muscle Area; SMI, Skeletal Muscle Index; VAT, Visceral Adipose Tissue; VATD, Visceral Adipose Tissue radiodensity; VATI, Visceral Adipose Tissue Index; SAT, Subcutaneous Adipose Tissue; SATD, Subcutaneous Adipose Tissue Radiodensity; SATI, Subcutaneous Adipose Tissue Index. Results are expressed as mean ± standard deviation or median [interquartile range]. Bold values indicate a significant *p*-value <0.05. *GLIM criteria is used for diagnosis of malnutrition.

### 3.5 Body composition and clinical outcomes in women

#### 3.5.1 BC in women considering length of hospital stay

As previously mentioned, distinct sex-related differences in length of hospital stay were observed, with women being less likely to remain hospitalized for more than 10 days compared to men.

Considering these results, when stratified women by length of hospital stay (<6 vs. ≥10 days; Table 7), those with prolonged hospitalization exhibited significantly lower Subcutaneous Adipose Tissue (SAT) area, percentage, radiodensity, and index (all *p* < 0.05). A nonsignificant trend toward higher Visceral Adipose Tissue (VAT) was also observed, whereas muscle parameters (LMM area, percentage, index and radiodensity) did not differ between the two groups.

Patients with prolonged hospital stay had more postoperative complications (*p* < 0.001), together with a trend toward higher mortality (*p* = 0.076) and a greater prevalence of rectal cancer (*p* = 0.07). Age, BMI, and cancer stage did not differ significantly.

#### 3.5.2 BC in women considering presence of postoperative complications

Women’s body composition parameters assessed by CT according to the presence of postoperative complications can be found in Supplementary Table 2. No significant differences in body composition were observed between women with or without complications.

#### 3.5.3 BC in women considering disease stage

Women BC parameters assessed by CT according to disease stage can be found in Supplementary Table 3. No significant differences in body composition were observed between women according to disease stage.

### 3.6 Comparative analysis of body composition in stage I–III by sex

Men with IV disease stage presented higher rates of postoperative complications compared to the same stage for women (50% vs. 15%, *p* = 0.045) as well as higher inflammatory burden in adipose tissue (VAT HU −90.07 ± 10.21 vs. − 99.15 ± 10.36; *p* = 0.013). Considering these results (found in Supplementary Table 1), we next restricted the analysis to patients with stage I–III colorectal cancer in order to minimize the influence of tumor burden on body composition (Table 8).

**Table 8:** Body composition parameters by CT by sex considering I-III cancer stages.

*n* = 274		Men (*n* = 174)	Women (*n* = 100)	*p* value
CT BC parameters				
Lean muscle	LMMA (cm^2^)	129.64 ± 26.18	90.97 ± 16.83	**<0.001**
	LMMA (%)	17.55 ± 4.43	14.12 ± 3.95	**<0.001**
	LMMI (cm2/m^2^)	44.69 ± 8.72	36.24 ± 6.1	**<0.001**
	LMD (HU)	40.63 ± 9.77	37.59 ± 9.61	**0.013**
Intermuscular adipose tissue	IMAT (cm^2^)	13.63 [9.04, 24.04]	14.76 [10.08, 22.29]	0.638
	IMAT (%)	1.92 [1.29, 3.0]	2.25 [1.55, 3.3]	0.076
	IMATD (HU)	−65.64 ± 6.48	−65.88 ± 6.49	0.775
	IMATI (cm2/m^2^)	4.78 [3.22, 8.3]	6.19 [3.97, 8.77]	**0.025**
Visceral adipose tissue	VAT (cm^2^)	212.26 ± 112.45	139.73 ± 79.88	**<0.001**
	VAT (%)	26.94 [18.76, 33.42]	20.61 [13.04, 25.95]	**<0.001**
	VATD (HU)	−94.8 ± 7.94	−93.21 ± 8.96	0.129
	VATI (cm^2^/m^2^)	73.68 ± 40.2	55.84 ± 31.78	**<0.001**
Subcutaneous adipose tissue	SAT (cm^2^)	160.8 ± 69.1	224.95 ± 119.4	**<0.001**
	SAT (%)	20.82 [16.31, 24.66]	31.27 [24.31, 38.45]	**<0.001**
	SATD (HU)	−98.41 [−104.81, −93.11]	−101.35 [−106.28, −96.6]	**0.014**
	SATI (cm^2^/m^2^)	52.71 [38.82, 67.86]	79.84 [55.27, 116.32]	**<0.001**
Demographic and clinical data				
Age		68.63 ± 10.97	68.94 ± 9.97	0.814
BMI		26.89 ± 4.16	26.98 ± 5.56	0.88
Weight loss	**(%)**	1.68 [0.0, 6.1]	2.07 [0.0, 7.32]	0.219
Malnutrition*	n(%)	59 (34)	39 (39)	0.474
Post-operative complications	n(%)	36 (21)	23 (23)	0.768
Exitus	n(%)	15 (9)	6 (6)	0.583

CT, Computed Tomography; HU, Hounsfield Units; LMMA, Lean Muscle Mass Area; LMMI, Lean Muscle Mass Index; LMD, Lean Muscle Radiodensity; IMAT, InterMuscular Adipose Tissue; IMATD, InterMuscular Adipose Tissue Radiodensity; IMATI, InterMuscular Adipose Tissue Index; VAT, Visceral Adipose Tissue; VATD, Visceral Adipose Tissue radiodensity; VATI, Visceral Adipose Tissue Index; SAT, Subcutaneous Adipose Tissue; SATD, Subcutaneous Adipose Tissue Radiodensity; SATI, Subcutaneous Adipose Tissue Index. Results are expressed as mean ± standard deviation or median [interquartile range]. *GLIM criteria is used for diagnosis of malnutrition. Bold values indicate a significant *p*-value <0.05.

In this subgroup, men consistently presented higher lean mass metrics, including LMMA, LMMI, and radiodensity, compared with women, despite having similar BMI (26.9 ± 4.7 kg/m^2^; *p* = 0.880) and age (68.7 ± 10.6 years; *p* = 0.814). Conversely, women displayed significantly greater Subcutaneous Adipose Tissue (SAT) across all measures (area, %, radiodensity, and index; all *p* < 0.05), as well as a higher IMAT index (*p* = 0.025). Visceral Adipose Tissue remained higher in men, as reflected by VATI (*p* < 0.001). No differences by sex were observed in weight loss considering I-III cancer stages.

### 3.7 Body composition-related clinical outcomes by sex

Despite the marked sex-related differences in body composition, no significant differences were observed in postoperative outcomes as reported previously in Table 8. The prevalence of malnutrition according to GLIM criteria was similar in men and women (36%; *p* = 0.474). Postoperative complications occurred in 22% of patients (*p* = 0.768), with comparable median hospital stay (5 [3.0–7.0] vs. 5 [3.0–7.0] days; *p* = 0.884) and 6-month mortality (8%; *p* = 0.583).

On the other hand, Table 9 presents the prevalence of low muscle mass, low muscle radiodensity, and high subcutaneous and visceral adipose tissue by sex in our sample, based on established cut-off points. Notably, although a high proportion of patients exhibited these adverse body composition features, current cut-offs may not capture the prognostic impact of sex-specific body composition phenotypes.

**Table 9:** Clinical outcomes by sex in stage I–III cancer patients according to established cut-off points for low muscle mass, low radiodensity, and high adipose tissue.

*n* = 274		Men (*n* = 174)	Women (*n* = 100)	*p* value
Low SMI, Prado 2008 (26)	n(%)	143 (82)	65 (65)	**0.002**
Low SMI, Martin 2013 (27)	n(%)	143 (82)	77 (77)	0.378
Low SMI, Dolan 2019 (28)	n(%)	127 (73)	75 (75)	0.825
Low SMD, Martin 2013 (27)	n(%)	111 (64)	78 (78)	**0.021**
High SAT, Doyle 2013 (29)	n(%)	107 (61)	36 (36)	**0.016**
High VAT, Doyle 2013 (29)	n(%)	106 (61)	76 (76)	**<0.001**

IR, interquartile range; SMI, Skeletal Muscle Index; SMD, Skeletal Muscle Radiodensity; VAT, isceral Adipose Tissue; SAT, Subcutaneous Adipose Tissue. Bold values indicate a significant *p*-value <0.05.

### 3.8 Comparative analysis of body composition by age

As current cut-off points do not account for age-related changes in body composition, we stratified the analysis by both sex and age to better capture these variations.

As expected, older patients exhibited lower muscle indices and radiodensity, along with higher adipose tissue levels in both sexes (Table 10).

**Table 10:** Age composition parameters by CT according to sex.

*n* = 274	Sex	≤49 y (M = 6; *F* = 2)	50–59 y (M = 26; *F* = 14)	60–69 y (M = 63; *F* = 36)	70–79 y (M = 51; *F* = 32)	>80 y (M = 28; *F* = 16)	*p* value
LMD (HU)	Male	50.69 ± 9.35	47.77 ± 9.07	46.04 ± 9.1	43.66 ± 7.45	39.39 ± 7.01	**<0.001**
	Female	31.03 ± 2.73	36.55 ± 3.99	37.02 ± 6.04	36.45 ± 6.3	34.46 ± 7.47	0.488
LMMI (cm^2^/m^2^)	Male	46.68 ± 7.72	45.8 ± 8.29	43.04 ± 9.53	37.64 ± 8.61	34.58 ± 9.41	**<0.001**
	Female	49.22 ± 5.15	46.23 ± 9.72	35.9 ± 8.16	37.24 ± 8.88	33.07 ± 9.52	**<0.001**
IMATD (HU)	Male	−62.45 ± 6.14	−63.71 ± 6.46	−65.69 ± 6.37	−65.68 ± 6.66	−67.97 ± 6.04	0.115
	Female	−60.82 ± 21.23	−63.53 ± 8.77	−65.57 ± 5.31	−66.01 ± 6.35	−68.99 ± 3.59	0.191
IMATI (cm^2^/m^2^)	Male	2.87 ± 1.47	3.77 ± 2.66	5.6 ± 4.12	6.51 ± 3.51	9.91 ± 5.0	**<0.001**
	Female	3.82 ± 4.53	4.02 ± 2.6	6.58 ± 2.83	7.3 ± 4.29	9.74 ± 5.07	**0.049**
VATD (HU)	Male	−93.3 ± 8.86	−93.8 ± 8.37	−94.96 ± 7.8	−95.31 ± 8.52	−94.78 ± 6.93	0.932
	Female	−82.04 ± 23.9	−89.31 ± 10.64	−93.72 ± 9.42	−93.96 ± 6.89	−95.38 ± 7.15	0.595
VATI (cm^2^/m^2^)	Male	46.72 ± 27.92	62.28 ± 42.77	68.55 ± 39.81	89.97 ± 41.1	71.93 ± 29.94	**0.006**
	Female	23.1 ± 32.65	34.25 ± 33.35	58.3 ± 31.67	64.75 ± 30.35	55.5 ± 25.05	**0.02**
SATD (HU)	Male	−99.86 ± 7.07	−97.0 ± 13.25	−98.13 ± 7.81	−97.17 ± 12.58	−97.96 ± 8.51	0.959
	Female	−81.76 ± 37.55	−98.79 ± 9.65	−99.04 ± 11.14	−100.88 ± 10.98	−101.67 ± 5.59	0.168
SATI (cm^2^/m^2^)	Male	57.27 ± 20.0	53.08 ± 24.26	56.59 ± 27.69	57.21 ± 24.68	52.2 ± 15.66	0.883
	Female	68.99 ± 93.9	76.9 ± 52.57	98.2 ± 50.32	88.85 ± 44.92	87.56 ± 37.81	0.636

LMMI, Lean Muscle Mass Index; LMD, Lean Muscle Radiodensity; IMATD, InterMuscular Adipose Tissue Radiodensity; IMATI, InterMuscular Adipose Tissue Index; VATD, Visceral Adipose Tissue Radiodensity; VATI, Visceral Adipose Tissue Index; SATD, Subcutaneous Adipose Tissue Radiodensity; SATI, Subcutaneous Adipose Tissue Index. Bold values indicate a significant *p*-value <0.05.

**Figure 2:**
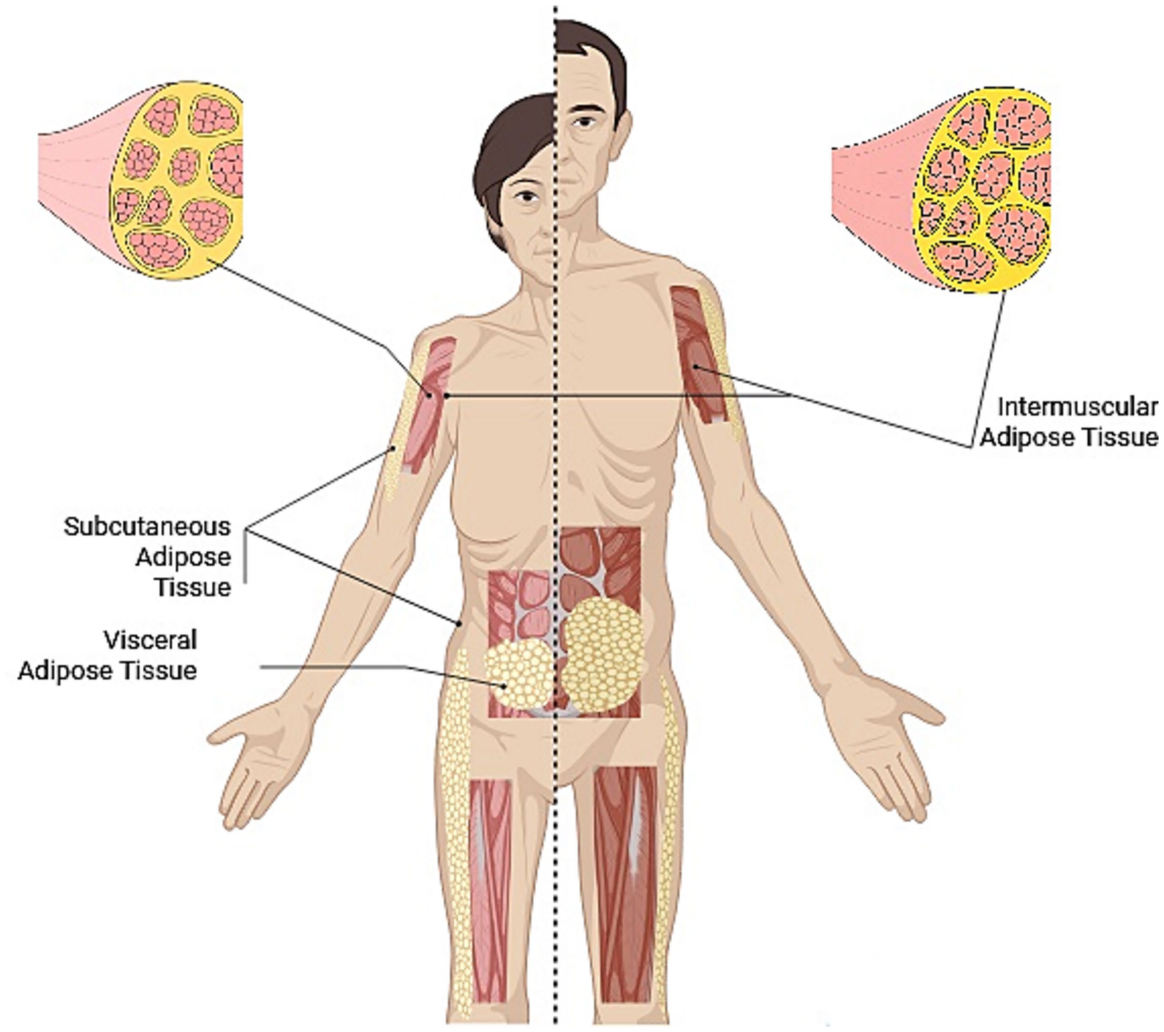
Phenotypic illustration of differences in body composition measured through TC according to sex patients with CRC prior to elective surgery.

The observed differences in body composition between men and women in CRC patients prior to elective surgery are illustrated in Figure 2, showing phenotypic variations measured by CT imaging.

## 4 Discussion

This study provides new evidence on sex-specific body composition phenotypes in patients undergoing colon cancer surgery. These results highlight how differences in muscle and adipose tissue distribution assessed by CT were observed according to postoperative outcomes, underscoring the importance of both qualitative and quantitative parameters.

Consistent with previous literature, men had greater skeletal muscle mass and visceral adiposity, whereas women had greater subcutaneous and intermuscular adipose tissue (40). Critically, these differences were not only descriptive but also reflected distinct patterns according to clinical outcomes. In women, lower SAT was consistently observed among prolonged hospital stay and complications, supporting the hypothesis that peripheral fat depots act as protective metabolic reserves in the perioperative period (41, 42). Multiple studies have demonstrated that SAT serves as a critical energy reserve and is associated with favorable outcomes in surgical and critical illness settings. Specifically, in female patients with cirrhosis, lower SAT index was independently associated with higher mortality, suggesting that depletion of subcutaneous fat reflects loss of a major metabolic reserve and leads to poor clinical outcomes (43). Similarly, in patients undergoing cardiovascular surgery, higher subcutaneous fat composition was associated with reduced all-cause and cardiac-cause mortality, while visceral fat was not predictive of outcomes (44). In men, patterns characterized by lower muscle quality (lower lean muscle radiodensity) as well as muscle quantity, and higher visceral adiposity were more frequently observed among those with adverse outcomes, emphasizing that both muscle quantity and quality are relevant in this group (45–47). These patterns were consistent across all age groups and tumor stages, reinforcing the robustness of sex-specific phenotypes.

Radiodensity, as a marker of tissue quality, emerged as a strong predictor of outcomes (27). This is consistent with growing evidence in other oncology settings that muscle attenuation provides additional prognostic information beyond simple muscle mass (47–49). In our cohort, lower preoperative Hounsfield units reflected poorer muscle quality and were more frequently observed in patients who experienced complications, often alongside reduced muscle mass. Similarly, increased HU values in adipose tissue suggested fibrotic or inflammatory remodeling, indicating early qualitative changes that may precede overt tissue depletion (50, 51).

Interestingly, our cohort challenges common assumptions regarding colorectal cancer patients (52). Among patients with advanced tumor stage (IV), individuals were younger and did not meet GLIM criteria for malnutrition, despite weight loss (8). This suggests that weight loss may serve as an early indicator of nutritional risk before formal diagnostic thresholds are reached. Body composition patterns in patients with advanced tumor stage (IV) did not appear worse than those in earlier stages (I–III), possibly reflecting timely diagnosis through screening and surgical referral before cachexia develops. However, they showed a tendency toward lower adipose tissue quantity and higher radiodensity in both SAT and VAT. This pattern may reflect early qualitative changes in adipose tissue, such as inflammatory or fibrotic remodeling, occurring before measurable losses in muscle mass or fat area. Although our sample size in advanced stages was limited, this finding reinforces the concept that qualitative alterations in adipose tissue may be among the earliest detectable metabolic disruptions in cancer progression.

Taken together, these findings illustrate that tumor stage alone is not sufficient to define metabolic and clinical risk, and that body composition phenotyping provides substantial discriminatory power.

Interestingly, when applying some established cut-offs, women in our cohort appeared to have higher rates of sarcopenia compared to Prado et al. (26), and greater myosteatosis compared to Martin et al. (27), a higher VAT compared to Doyle et al. (29), whereas men showed higher SAT compared to Doyle et al. (29). However, our own analyses highlighted a different pattern: adverse outcomes in men were mainly driven by low muscle quality and excess VAT, while in women SAT depletion emerged as the predominant risk factor. In line with this, age-stratified analyses further emphasized that body composition variability should be interpreted not only by sex but also by age, highlighting the need for refined phenotyping strategies. These differences underscore the limitations of uniform cutoff values and support the need for context-specific, sex- and age-adjusted thresholds that integrate both muscle quality and adipose tissue distribution.

The clinical implications are clear. Opportunistic CT in oncology provides highly accurate information on tissue quantity, quality, and distribution, allowing for the development of phenotypic profiles that can improve perioperative risk stratification and guide personalized nutritional strategies.

Recent advances have proposed ultrasound and other non-radiological techniques as potential alternatives for assessing muscle mass and quality in oncology patients. However, most of these approaches rely on single-muscle measurements and show limited validation against CT-based parameters or clinical outcomes (53–57). In this context, our findings reinforce the value of CT-derived assessment, which uniquely integrates tissue quality and adipose distribution, providing a more comprehensive reflection of the patient’s metabolic and functional reserve.

This study has some limitations. Its retrospective design precludes causal inference, and although multicenter and relatively large, the number of patients with advanced stages was limited. Only patients undergoing elective laparoscopic surgery were included; therefore, the findings may not be generalizable to those receiving open or emergency procedures. Data on (neo)adjuvant chemotherapy, which could also influence body composition, were not available. Otherwise, only bivariate comparisons are reported. Further studies adjusting for potential confounders such as BMI, cancer stage, age, and sex are required to confirm these associations and better elucidate the independent contribution of each body composition parameter to postoperative outcomes. Despite these limitations, the standardized CT methodology, multicenter design, and consistent patterns observed in muscle and adipose compartments support the validity of our findings.

This study provides new evidence on sex-specific body composition phenotypes in patients undergoing surgery for colon cancer. These results highlight the patterns of muscle and adipose tissue distribution assessed by CT in relation to postoperative outcomes. To our knowledge, this is one of the first studies to stratify both lean and adipose compartments by sex and age, offering insight into how tissue phenotypes may differentially influence metabolic and clinical risk.

## 5 Conclusion

In this multicenter cohort of colorectal cancer patients, preoperative CT-derived body composition parameters, encompassing both muscle and adipose compartments, differed according to surgical outcomes. These findings highlight the importance of nutritional assessment that considers not only muscle mass but also muscle quality and fat distribution, thereby complementing current GLIM criteria and providing a more accurate reflection of metabolic reserve. Integrating CT-based body composition into clinical pathways may improve risk stratification and guide targeted nutritional and prehabilitation strategies. Future research should examine these patterns in larger and more diverse populations, across different tumor types, and establish clinically relevant sex- and age-specific cut-offs for routine use.

## Data Availability

The raw data supporting the conclusions of this article will be made available by the authors, without undue reservation.

## Footnotes

1
https://www.python.org/

## Glossary

BC: Body Composition
BMI: Body Mass Index
CRC: Colorectal Cancer
CT: Computed Tomography
GLIM: Global Leadership Initiative on Malnutrition
EWGSOP2: European Working Group on Sarcopenia in Older People 2
LMMA: Lean Muscle Mass Area
LMMI: Lean Muscle Mass Index
LMD: Lean Muscle Radiodensity
SMI: Skeletal Muscle Index
SMA: Skeletal Muscle Area
SMD: Skeletal Muscle Radiodensity
SAT: Subcutaneous Adipose Tissue
SATI: Subcutaneous Adipose Tissue Index
SATD: Subcutaneous Adipose Tissue Radiodensity
VAT: Visceral Adipose Tissue
VATI: Visceral Adipose Tissue Index
VATD: Visceral Adipose Tissue Radiodensity
IMAT: Intermuscular Adipose Tissue
IMATI: Intermuscular Adipose Tissue Index
IMATD: Intermuscular Adipose Tissue Radiodensity
FFMI: Fat Free Mass Index
BIA: Bioelectrical Impedance
IR: Interquartile Range
SD: Standard Deviation
