# Supplementary material for: Sex-specific CT-derived muscle and fat phenotypes in colon cancer: implications for nutritional and metabolic assessment
Source: Front Nutr. 2025 Nov 21;12:1728741. doi: 10.3389/fnut.2025.1728741 (PMC12678153; doi:10.3389/fnut.2025.1728741)
Supplement: Supplementary file 1 [file Table_1.DOCX]

**Table 1.** Body Composition parameters though CT by gender and stage IV cancer.

| **n=39** | | **Men (n=26)** | **Women (n=13)** | ***P value*** |
| --- | --- | --- | --- | --- |
| **CT BC parameters** | |  |  |  |
| Lean Muscle | LMMA (cm^2^) | 124.53±25.43 | 97.25±12.4 | **<0.001** |
|  | LMMA (%) | 18.33±4.12 | 15.05±5.2 | **0.038** |
|  | LMMI (cm2/m^2^) | 43.11±8.31 | 39.1±5.06 | 0.12 |
|  | LMD (HU) | 40.95±8.18 | 38.43±13.08 | 0.464 |
| Intermuscular Adipose  Tissue | IMAT (cm^2^) | 13.12±11.86 | 18.26±11.2 | 0.202 |
|  | IMAT (%) | 1.58 [1.1,2.18] | 2.07 [1.6,3.06] | **0.018** |
|  | IMATD (HU) | -61.6±6.56 | -65.05±7.55 | 0.15 |
|  | IMATI (cm2/m^2^) | 4.51±4.15 | 7.36±4.62 | 0.059 |
| Skeletal  Muscle | SMA (cm2) | 137.64±26.04 | 115.51±17.77 | **0.009** |
|  | SMA (%) | 20.12±3.66 | 17.54±4.85 | **0.07** |
|  | SMI (cm^2^/m^2^) | 47.63±8.3 | 46.46±7.27 | 0.67 |
|  | SMD (HU) | 31.36±14.15 | 23.04±17.86 | 0.121 |
| Visceral  Adipose  Tissue | VAT (cm^2^) | 145.68±82.83 | 203.76±104.96 | 0.067 |
|  | VAT (%) | 19.66±8.63 | 28.05±13.24 | **0.022** |
|  | VATD (HU) | -90.07±10.21 | -99.15±10.36 | **0.013** |
|  | VATI (cm^2^/m^2^) | 49.85±26.38 | 83.23±45.65 | **0.006** |
| Subcutaneous Adipose  Tissue | SAT (cm^2^) | 176.95±99.13 | 202.71±122.69 | 0.484 |
|  | SAT (%) | 23.59±9.01 | 27.22±13.36 | 0.321 |
|  | SATD (HU) | -89.52±12.43 | -96.0±12.34 | 0.132 |
|  | SATI (cm^2^/m^2^) | 61.16±33.57 | 82.27±50.48 | 0.127 |
| **Demographic and clinical data** | |  |  |  |
| Age |  | 65.08±11.1 | 59.23±9.22 | 0.111 |
| BMI |  | 25.54±3.92 | 28.11±5.36 | 0.096 |
| Malnutrition* | n(%) | 13 (50) | 3 (23) | 0.169 |
| Postoperative complications | n(%) | 13 (50) | 2 (15) | **0.045** |
| Exitus | n(%) | 5 (19) | 1 (8) | 0.643 |

^1^ Abbreviations: CT= Computed Tomography; HU=Hounsfield Units; LMMA: Lean Muscle Mass Area; LMMI=Lean Muscle Mass Index; LMD= Lean Muscle Radiodensity IMAT=InterMuscular Adipose Tissue; IMATD= InterMuscular Adipose Tissue Radiodensity; IMATI= InterMuscular Adipose Tissue Index; SMA= Skeletal Muscle Area; SMI: Skeletal Muscle Index; VAT= Visceral Adipose Tissue; VATD= Visceral Adipose Tissue radiodensity; VATI= Visceral Adipose Tissue Index; SAT= Subcutaneous Adipose Tissue; SATD= Subcutaneous Adipose Tissue Radiodensity; SATI= Subcutaneous Adipose Tissue Index; BMI= Body Mass Index. Results are expressed as mean ± standard deviation or median [interquartile range]. *GLIM criteria is used for diagnosis of malnutrition.. Bold values indicate a significant p-value <0.05.

**Table 2.** Women Body Composition parameters by CT according to presence of post-operative complication.

| **Women CT BC parameters (n=188)** | | **No post-op complication (n=138)** | **Presence of post-op complication (n=50)** | ***P value*** |
| --- | --- | --- | --- | --- |
| Lean Muscle | LMMA (cm^2^) | 91.69±15.99 | 89.49±16.33 | 0.408 |
|  | LMMA (%) | 14.49±3.9 | 14.6±4.18 | 0.869 |
|  | LMMI (cm2/m^2^) | 36.68±5.95 | 36.91±6.52 | 0.817 |
|  | LMD (HU) | 37.24±9.93 | 36.08±10.14 | 0.485 |
| Intermuscular Adipose  Tissue | IMAT (cm^2^) | 14.1 [9.3,20.82] | 12.93 [8.46,20.06] | 0.49 |
|  | IMAT (%) | 2.17 [1.46,3.02] | 1.96 [1.5,2.74] | 0.49 |
|  | IMATD (HU) | -63.79±6.66 | -62.81±6.21 | 0.624 |
|  | IMATI (cm2/m^2^) | 6.19 [3.97, 8.77] | 5.81 [4.01, 8.44] | 0.227 |
| Skeletal  Muscle | SMA (cm2) | 107.96±19.57 | 104.37±15.55 | 0.244 |
|  | SMA (%) | 16.34 [14.1,18.5] | 16.52[14.14,19.39] | 0.736 |
|  | SMI (cm^2^/m^2^) | 43.21±7.39 | 43.04±5.96 | 0.879 |
|  | SMD (HU) | 22.67±14.74 | 22.19±15.57 | 0.844 |
| Visceral  Adipose  Tissue | VAT (cm^2^) | 170.31±88.19 | 178.06±114.05 | 0.624 |
|  | VAT (%) | 25.24±12.04 | 25.32±11.89 | 0.965 |
|  | VATD (HU) | -96.65 [-101.19,-91.69) | -96.81 [-100.97,-86.2] | 0.618 |
|  | VATI (cm^2^/m^2^) | 68.74±36.68 | 72.9±44.29 | 0.517 |
| Subcutaneous Adipose  Tissue | SAT (cm^2^) | 188.07±120.18 | 161.77±103.3 | 0.171 |
|  | SAT (%) | 26.12±12.04 | 23.28±11.43 | 0.15 |
|  | SATD (HU) | -95.43±11.46 | -92.14±12.65 | 0.093 |
|  | SATI (cm^2^/m^2^) | 75.49±48.08 | 66.44±41.85 | 0.24 |

^1^ Abbreviations: CT= Computed Tomography; HU=Hounsfield Units; LMMA: Lean Muscle Mass Area; LMMI=Lean Muscle Mass Index; LMD= Lean Muscle Radiodensity IMAT=InterMuscular Adipose Tissue; IMATD= InterMuscular Adipose Tissue Radiodensity; IMATI= InterMuscular Adipose Tissue Index; SMA= Skeletal Muscle Area; SMI: Skeletal Muscle Index; VAT= Visceral Adipose Tissue; VATD= Visceral Adipose Tissue radiodensity; VATI= Visceral Adipose Tissue Index; SAT= Subcutaneous Adipose Tissue; SATD= Subcutaneous Adipose Tissue Radiodensity; SATI= Subcutaneous Adipose Tissue Index. Results are expressed as mean ± standard deviation or median [interquartile range]. Bold values indicate a significant p-value <0.05.

**Table 3** Women Body Composition parameters by CT according to disease stage.

| **Women (n=106)** | | **I-III stage (n=100)** | **IV stage(n=6)** | ***P value*** |
| --- | --- | --- | --- | --- |
| **CT BC parameters** | |  |  |  |
| Lean Muscle | LMMA (cm^2^) | 90.97±16.83 | 100.47±14.9 | 0.18 |
|  | LMMA (%) | 14.12±3.95 | 16.23±7.31 | 0.231 |
|  | LMMI (cm2/m^2^) | 36.24±6.1 | 40.46±6.06 | 0.103 |
|  | LMD (HU) | 37.59±9.61 | 40.84±15.66 | 0.44 |
| Intermuscular Adipose  Tissue | IMAT (cm^2^) | 17.41±10.6 | 21.2±15.48 | 0.41 |
|  | IMAT (%) | 2.25 [1.55,3.3] | 2.46 [1.56,3.96] | 0.702 |
|  | IMATD (HU) | -65.88±6.49 | -66.39±8.57 | 0.855 |
|  | IMATI (cm2/m^2^) | 6.91±4.04 | 8.57±6.45 | 0.346 |
| Skeletal  Muscle | SMA (cm2) | 108.37±20.31 | 121.67±19.77 | 0.122 |
|  | SMA (%) | 16.65±3.81 | 19.06±6.64 | 0.155 |
|  | SMI (cm^2^/m^2^) | 43.14±7.18 | 49.03±8.24 | 0.055 |
|  | SMD (HU) | 21.59±14.84 | 23.42±23.95 | 0.779 |
| Visceral  Adipose  Tissue | VAT (cm^2^) | 139.73±79.88 | 131.83±90.36 | 0.816 |
|  | VAT (%) | 20.07±11.08 | 16.48±8.96 | 0.439 |
|  | VATD (HU) | -93.21±8.96 | -94.19±13.39 | 0.802 |
|  | VATI (cm^2^/m^2^) | 54.84±31.78 | 53.84±37.11 | 0.882 |
| Subcutaneous Adipose  Tissue | SAT (cm^2^) | 224.95±119.4 | 278.6±130.9 | 0.29 |
|  | SAT (%) | 31.21±10.27 | 37.28±11.12 | 0.164 |
|  | SATD (HU) | -101.35 [-106.28,-96.6] | -103.24 [-110.92,-90.53] | **0.627** |
|  | SATI (cm^2^/m^2^) | 89.94±47.48 | 113.23±54.47 | 0.25 |
| **Demographic and clinical data** | |  |  |  |
| Age |  | 68.94±9.97 | 57.67±9.56 | **0.008** |
| BMI |  | 26.98±5.56 | 28.4±6.67 | 0.548 |
| Weight loss | **(%)** | 2.07 [0.0,7.32] | 0.0 [-0.63,3.13] | 0.144 |
| Malnutrition* | n(%) | 39 (39) | 1 (17) | 0.405 |
| Exitus | n(%) | 6 (6) | 0 (0) | 1 |

^1^ Abbreviations: CT= Computed Tomography; HU=Hounsfield Units; LMMA: Lean Muscle Mass Area; LMMI=Lean Muscle Mass Index; LMD= Lean Muscle Radiodensity IMAT=InterMuscular Adipose Tissue; IMATD= InterMuscular Adipose Tissue Radiodensity; IMATI= InterMuscular Adipose Tissue Index; SMA= Skeletal Muscle Area; SMI: Skeletal Muscle Index; VAT= Visceral Adipose Tissue; VATD= Visceral Adipose Tissue radiodensity; VATI= Visceral Adipose Tissue Index; SAT= Subcutaneous Adipose Tissue; SATD= Subcutaneous Adipose Tissue Radiodensity; SATI= Subcutaneous Adipose Tissue Index; BMI= Body Mass Index. Results are expressed as mean ± standard deviation or median [interquartile range]. *GLIM criteria is used for diagnosis of malnutrition.. Bold values indicate a significant p-value <0.05.
